# Supplementary material for: Molecular Insights into Diapause Mechanisms in Telenomus remus for Improved Biological Control
Source: Insects. 2025 Apr 8;16(4):393. doi: 10.3390/insects16040393 (PMC12027507; doi:10.3390/insects16040393)
Supplement: Supplementary file 1 [file insects-16-00393-s001.zip › insects-3429933-supplementary.pdf]

Supplementary Table S1: KEGG Pathway Analysis of Diapause-Associated Genes in Telenomus remus

Primary metabolism	Secondary metabolism	Tertiary metabolism	KO number of involvedgenes
Metabolism	Carbohydrate metabolism	Glycolysis / Gluconeogenesis	ko00010
		Citrate cycle (TCA cycle)	ko00020
		Pentose phosphate pathway	ko00030
		Pentose and glucuronate interconversions	ko00040
		Fructose and mannose metabolism	ko00051
		Galactose metabolism	ko00052
		Ascorbate and aldarate metabolism	ko00053
		Starch and sucrose metabolism	ko00500
		Amino sugar and nucleotide sugar metabolism	ko00520
		Inositol phosphate metabolism	ko00562
		Pyruvate metabolism	ko00620
		Glyoxylate and dicarboxylate metabolism	ko00630
		Propanoate metabolism	ko00640
		Butanoate metabolism	ko00650
	Lipid metabolism	Fatty acid biosynthesis	ko00061
		Fatty acid elongation	ko00062
		Fatty acid degradation	ko00071
		Cutin, suberine and wax biosynthesis	ko00073
		Steroid biosynthesis	ko00100
		Primary bile acid biosynthesis	ko00120
		Steroid hormone biosynthesis	ko00140
		Glycerolipid metabolism	ko00561
		Glycerophospholipid metabolism	ko00564
		Ether lipid metabolism	ko00565
		Arachidonic acid metabolism	ko00590
		Linoleic acid metabolism	ko00591
		alpha-Linolenic acid metabolism	ko00592
		Sphingolipid metabolism	ko00600
		Biosynthesis of unsaturated fatty acids	ko01040
	Energy metabolism	Oxidative phosphorylation	ko00190
		Methane metabolism	ko00680
		Carbon fixation in photosynthetic organisms	ko00710
		Carbon fixation pathways in prokaryotes	ko00720
		Nitrogen metabolism	ko00910
		Sulfur metabolism	ko00920
	Amino acid metabolism	Arginine biosynthesis	ko00220

		Alanine, aspartate and glutamate metabolism	ko00250
		Glycine, serine and threonine metabolism	ko00260
		Cysteine and methionine metabolism	ko00270
		Valine, leucine and isoleucine degradation	ko00280
		Valine, leucine and isoleucine biosynthesis	ko00290
		Lysine biosynthesis	ko00300
		Lysine degradation	ko00310
		Arginine and proline metabolism	ko00330
		Histidine metabolism	ko00340
		Tyrosine metabolism	ko00350
		Phenylalanine metabolism	ko00360
		Tryptophan metabolism	ko00380
		Phenylalanine, tyrosine and tryptophan biosynthesis	ko00400
	Nucleotide metabolism	Purine metabolism	ko00230
		Pyrimidine metabolism	ko00240
Genetic Information Processing	Translation	Aminoacyl-tRNA biosynthesis	ko00970
		Ribosome	ko03008、ko03010
		Nucleocytoplasmic transport	ko03013
		mRNA surveillance pathway	ko03015
	Transcription	Aminoacyl-tRNA biosynthesis	ko00970
		RNA polymerase	ko03020
		Basal transcription factors	ko03022
		Spliceosome	ko03040
	Replication and repair	DNA replication	ko03030
		Base excision repair	ko03410
		Nucleotide excision repair	ko03420
		Mismatch repair	ko03430
		Homologous recombination	ko03440
		Non-homologous end-joining	ko03450
		Fanconi anemia pathway	ko03460
	Folding, sorting and degradation	Ubiquitin mediated proteolysis	ko04120
		Protein processing in endoplasmic reticulum	ko04141
		SNARE interactions in vesicular transport	ko04130
		Proteasome	ko03050
		RNA degradation	ko03018
		Protein export	ko03060
		Sulfur relay system	ko04122
		SNARE interactions in vesicular transport	ko04130
Environmental Information Processing	Membrane transport	ABC transporters	ko02010

		Bacterial secretion system	ko03070
	Signal transduction	Two-component system	ko02020
		MAPK signaling pathway	ko04010、ko04011 ko04013、ko04016
		ErbB signaling pathway	ko04012
		Ras signaling pathway	ko04014
		Rap1 signaling pathway	ko04015
		Calcium signaling pathway	ko04020
		cGMP-PKG signaling pathway	ko04022
		cAMP signaling pathway	ko04024
		HIF-1 signaling pathway	ko04066
		FoxO signaling pathway	ko04068
		Phosphatidylinositol signaling system	ko04070
		Phospholipase D signaling pathway	ko04072
		PI3K-Akt signaling pathway	ko04151
		AMPK signaling pathway	ko04152
		NF-kappa B signaling pathway	ko04064
		HIF-1 signaling pathway	ko04066
		Sphingolipid signaling pathway	ko04071
		mTOR signaling pathway	ko04150
		Wnt signaling pathway	ko04310
		Notch signaling pathway	ko04330
		Hedgehog signaling pathway	ko04340 ko04341
		TGF-beta signaling pathway	ko04350
		Apelin signaling pathway	ko04371
		Hippo signaling pathway	ko04390、ko04391 ko04392
		JAK-STAT signaling pathway	ko04630
		TNF signaling pathway	ko04668
	Signaling molecules and interaction	Cytokine-cytokine receptor interaction	ko04060
		Neuroactive ligand-receptor interaction	ko04080
		Cell adhesion molecules	ko04514
		ECM-receptor interaction	ko04512
Cellular Processes	Transport and catabolism	Autophagy	ko04136、ko04138 ko04140
		Mitophagy	ko04137 ko04139
		Lysosome	ko04142
		Endocytosis	ko04144
		Phagosome	ko04145
		Peroxisome	ko04146
	Cell growth and death	Apoptosis	ko04210、ko04214 ko04215
		Necroptosis	ko04217
		Cellular senescence	ko04218
		Oocyte meiosis	ko04114
		Cell cycle	ko04110、ko04111 ko04112

		Meiosis - yeast	ko04113
		p53 signaling pathway	ko04115
		Ferroptosis	ko04216
	Cell motility	Regulation of actin cytoskeleton	ko04810
	Cellular community	Quorum sensing	ko02024
		Biofilm formation - Escherichia coli	ko02026
		Focal adhesion	ko04510
		Adherens junction	ko04520
		Tight junction	ko04530
		Gap junction	ko04540
		Signaling pathways regulating pluripotency of stem cells	ko04550
Organismal Systems	Immune system	Chemokine signaling pathway	ko04062
		Complement and coagulation cascades	ko04610
		Platelet activation	ko04611
		Antigen processing and presentation	ko04612
		Neutrophil extracellular trap formation	ko04613
		receptor signaling pathway	ko04620、ko04621 ko04622、ko04660 ko04662、ko04625
		signaling pathway	ko04664、ko04657
		Cytosolic DNA-sensing pathway	ko04623
		Toll and Imd signaling pathway	ko04624
		Hematopoietic cell lineage	ko04640
		Natural killer cell mediated cytotoxicity	ko04650
		Fc gamma R-mediated phagocytosis	ko04666
		Leukocyte transendothelial migration	ko04670
		Th1 and Th2 cell differentiation	ko04658、ko04659
	Endocrine system	PPAR signaling pathway	ko03320
		Renin-angiotensin system	ko04614
		Insulin signaling pathway	ko04910
		Insulin secretion	ko04911
		GnRH signaling pathway	ko04912
		Ovarian steroidogenesis	ko04913
		Progesterone-mediated oocyte maturation	ko04914
		Estrogen signaling pathway	ko04915
		Melanogenesis	ko04916
		Prolactin signaling pathway	ko04917
		Thyroid hormone synthesis	ko04918
		Thyroid hormone signaling pathway	ko04919
		Adipocytokine signaling pathway	ko04920

	Oxytocin signaling pathway	ko04921
	Glucagon signaling pathway	ko04922
	Regulation of lipolysis in adipocytes	ko04923
	Renin secretion	ko04924
	Aldosterone synthesis and secretion	ko04925
	Relaxin signaling pathway	ko04926
	Cortisol synthesis and secretion	ko04927
	hormone synthesis, secretion and action	ko04928 ko04935
	GnRH secretion	ko04929
Circulatory system	Cardiac muscle contraction	ko04260
	Adrenergic signaling in cardiomyocytes	ko04261
	Vascular smooth muscle contraction	ko04270
Digestive system	Digestive system	ko04970
	Gastric acid secretion	ko04971
	Pancreatic secretion	ko04972
	Carbohydrate digestion and absorption	ko04973
	Protein digestion and absorption	ko04974
	Fat digestion and absorption	ko04975
	Bile secretion	ko04976
	Vitamin digestion and absorption	ko04977
	Mineral absorption	ko04978
	Cholesterol metabolism	ko04979
Nervous system	Long-term potentiation	ko04720
	Synaptic vesicle cycle	ko04721
	Neurotrophin signaling pathway	ko04722
	Retrograde endocannabinoid signaling	ko04723
	Glutamatergic synapse	ko04724
	Cholinergic synapse	ko04725
	Serotonergic synapse	ko04726
	GABAergic synapse	ko04727
	Dopaminergic synapse	ko04728
	Long-term depression	ko04730
Sensory system	Olfactory transduction	ko04740
	Taste transduction	ko04742
	Phototransduction	ko04744、ko04745
	Inflammatory mediator regulation of TRP channels	ko04750
Environmental adaptation	Plant-pathogen interaction	ko04626
	Circadian rhythm	ko04710、ko04711 ko04712
	Circadian entrainment	ko04713
	Thermogenesis	ko04714
Development and regeneration	Dorso-ventral axis formation	ko04320
	Axon	ko04360、ko04361
	Osteoclast differentiation	ko04380

Aging	Longevity regulating pathway	ko04211、ko04212 ko04213
